# Outbreak of Central American born *Shigella sonnei* in two youth camps in Belgium in the summer of 2019

**DOI:** 10.1007/s10096-021-04164-y

**Published:** 2021-02-11

**Authors:** An Van den Bossche, Pieter-Jan Ceyssens, Sarah Denayer, Naïma Hammami, Maaike van den Beld, Timothy J. Dallman, Wesley Mattheus

**Affiliations:** 1 Scientific Service Bacterial Diseases - Infectious Diseases in Humans, Sciensano, Brussels, Belgium; 2 National Reference Center of Salmonella and Shigella - Infectious Diseases in Humans, Sciensano, Brussels, Belgium; 3 Scientific Service Food Pathogens - Infectious Diseases in Humans, Sciensano, Brussels, Belgium; 4 Team Infection Prevention, Flemish Agency for Care and Health, Ghent, Belgium; 5 Infectious Disease Research, Diagnostics and Laboratory Surveillance, Centre for Infectious Disease Control, National Institute for Public Health and the Environment, Bilthoven, The Netherlands; 6 National Infection Service, Public Health England, London, UK

**Keywords:** *Shigella sonnei*, Outbreak, Next-generation sequencing, Cluster analyses

## Abstract

In 2019, an outbreak of *Shigella sonnei* occurred during two youth camps in Belgium. The clustering of isolates from both camps was confirmed by next-generation sequencing, as well as a secondary infection of a technician. The outbreak strain clustered with internationally isolated strains from patients with recent travel history to Central America. This report exemplifies enhanced surveillance and international collaboration between public health institutes by enabling to link local outbreaks to region-specific sublineages circulating abroad.

**Supplementary Information:**

The online version contains supplementary material available at 10.1007/s10096-021-04164-y.

## Introduction

In Belgium, around 400 human Shigellosis cases are confirmed by the National Reference Centre (NRC) for *Salmonella* and *Shigella* each year. As in most developed countries [1, 2], *Shigella sonnei* is the most frequently reported species representing 76.5% of the received isolates in 2019. Most cases are reported as isolated cases. However, here we report an *S. sonnei* outbreak in two youth camps during the summer of 2019, which was further analysed by next-generation-sequencing (NGS) for phylogenetic clustering and source tracking.

## Onset and epidemiology of the outbreak

**Fig. 1:**
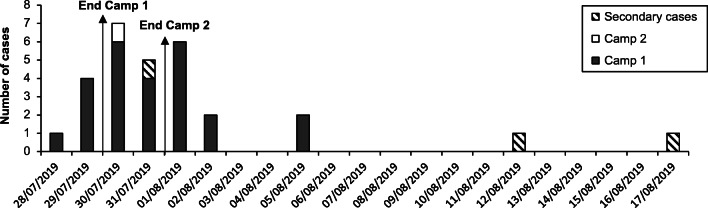
Epidemiological curve of all confirmed *S. sonnei* cases. Overview of the confirmed *S. sonnei* cases of both camps and secondary cases, sorted by date of sampling

On July 30th 2019, the Public Health and Surveillance Department was informed of a possible *Shigella* outbreak in a youth camp that took place in the Luxembourg province from July 19th to July 29th. In total, 47 (37 children and 10 adults) of the 162 (29.0%) participants were reported having clinical symptoms associated with a *Shigella* infection by the end of the camp. For 25/47 (53.2%) cases, the isolation of *S. sonnei* by local laboratories was confirmed during the subsequent days (Fig. 1; Online Resource 1). One week later, there was a notification of a gastro-intestinal outbreak in a second youth camp localized nearby, taking place from July 21st to July 31st. At least 20 children were ill, and for one person, *S. sonnei* was isolated from stool. During August, three secondary cases were reported and confirmed, being two parents of youth camp 1 and a technician of a local clinical laboratory.

## Epidemiological and source investigation

Since the authorities were only notified at or after the end of both camps, no interventions were implemented on site, and sampling of possible sources of infection could only be initiated after both camps were finished. Two types of sampling were organized. First, leftovers of meals consumed at camp 1 (including spaghetti sauce, soup, potatoes, apples, pears and onion) were investigated for the presence of *S. sonnei*. Secondly, water samples were taken in the environment, since both camps used water that was delivered by the same farmer. However, for both series of samples, *Shigella* was not detected.

Prior to youth camp 1, two children were already suffering from diarrhoea but were not treated by antibiotics. One of these, who was experiencing bloody diarrhoea, had returned from a trip to Guatemala one day before joining the camp. A stool sample taken on the 5th of August was negative. However, the child was not having symptoms anymore at the moment of sampling. Therefore, it was hypothesized that this person might be the index case who introduced *S. sonnei* into the first camp. Since members of camp 1 visited camp 2 and no *Shigella* could be isolated from food or water samples, it is likely that the infection was transmitted between camp participants by human-to-human contacts. Both camps were completely self-sustainable tent camps, including self-build toilets and showers. This may lead to poor hygiene, facilitating transmission of the contagious microorganisms, such as the low-dose infectious *Shigella sonnei* between the participants.

## Microbial characterization and molecular clustering of the human isolates

All confirmed human isolates were sent to the NRC for *Salmonella* and *Shigella*, where the *S. sonnei* species was reconfirmed by classical slide agglutination for all isolates. Subsequently, seven isolates linked to camp 1, the isolate associated with camp 2 and the strain isolated from the laboratory technician were selected for molecular analyses by NGS. DNA was extracted by semi-automated DNA extraction using the Bacterial DNA Kit (MagCore). DNA libraries were prepared using the Nextera XT DNA Sample Preparation Kit (Illumina) and sequenced on a Illumina MiSeq. Fastq files were further processed as described by Ventola et al. 2019 [3].

The selected strains were compared by single-nucleotide polymorphism (SNP) analyses using the CLC Bio Genome Workbench (RRID:SCR_011853). Reads were mapped against the reference genome *S. sonnei* Ss046 (CP000038.1), and SNPs were called with minimal coverage of 10, minimal count of 10 and minimal frequency of 0.7. No SNP differences were detected (maximum likelihood substitution model, Jukes-Cantor 2.0) between all 7 isolates of cases of camp 1, the single isolate of camp 2 or the isolate from the laboratory technician. This close clustering confirmed the presence of a single outbreak in camp 1, that camp 2 was infected with the same outbreak strain and indicates that the laboratory technician was infected during manipulation of the outbreak samples.

## Clustering with internationally isolated *S. sonnei* strains

**Fig. 2:**
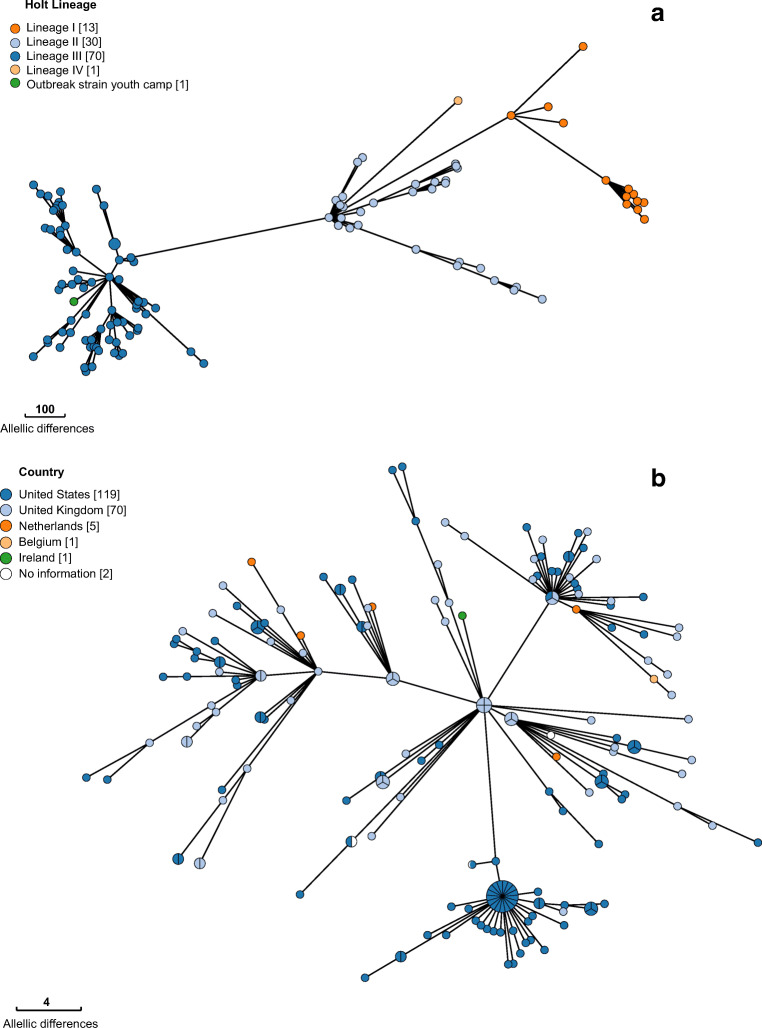
Minimum Spanning Trees using cgMLST data (EnteroBase). **a** MST of the outbreak strain and a collection of strains published by Holt et al. [4]. Each node represents an isolate, with different node colours indicating the phylogenetic Holt lineage. **b** MST of the HC10_463. Each node represents an isolate, with different node colours indicating the country of isolation

In 2012, Holt and colleagues defined four lineages (I to IV) within *S. sonnei*, based on SNP analyses on 132 globally distributed isolates [4], and a fifth lineage from Latin America and Africa was recently added [5]. cgMLST analyses, using the 2513 loci schema of the EnteroBase database (cgMLST V1 + HierCC V1, RRID:SCR_019019) [6], on this strain collection demonstrate that the outbreak strain can be phylogenetically classified as part of the Holt lineage III (Fig. 2a). This lineage is globally widespread over all continents, in contrast to lineage I and II which are mainly detected in Europe. The closest match within this collection could however only be found at 88 loci differences.

Subsequently, the outbreak strain was compared with all publicly available *Shigella* strains in EnteroBase (cgMLST V1 + HierCC V1). Using EnteroBase nomenclature, the outbreak strain has the cgMLST sequence type ST110297 and is classified in the hierarchical cluster (HC10) 463. A Minimum Spanning Tree (MST) of HC10_463, which contains isolates with maximum 10 pair-wise allelic differences (AD) [7], is shown in Fig. 2b. A cluster of 32 *S. sonnei* isolates was identified within the HC10_463 containing the outbreak strain, strains isolated from the USA, UK and the Netherlands. The closest match of 1 allelic difference was found with a strain isolated in the UK in 2019. Secondly, a strain isolated in the Netherlands in 2017 was detected at 6 AD.

**Fig. 3:**
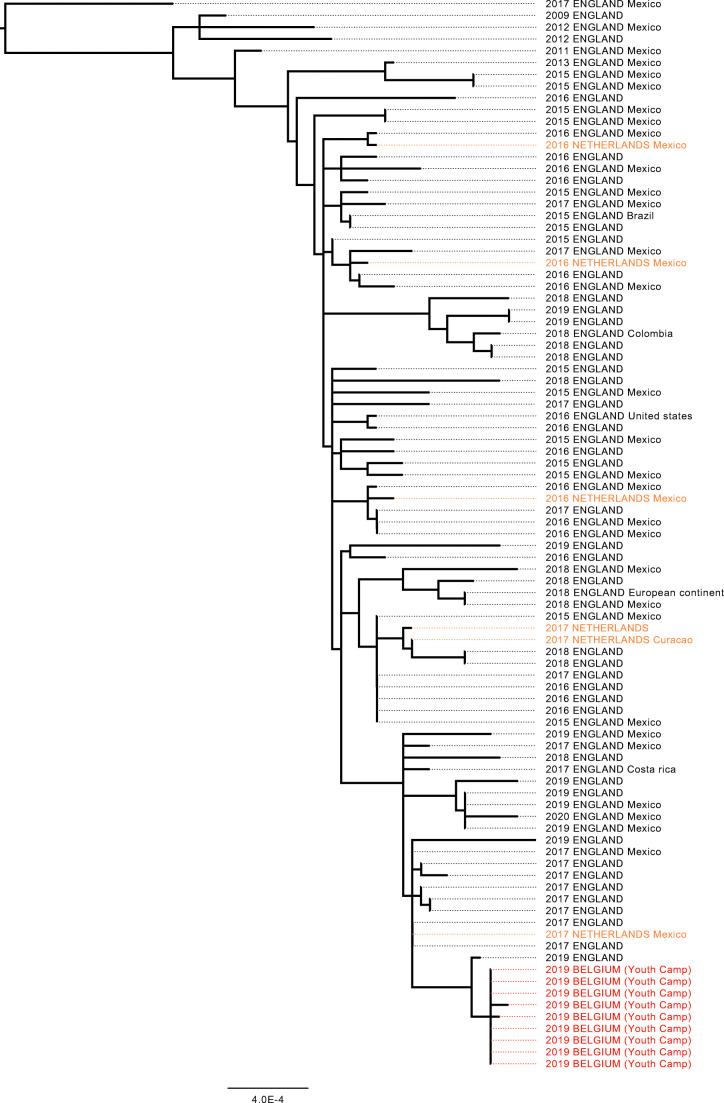
Twenty-five SNP single-linkage cluster of isolates from Belgium, UK and the Netherlands. Maximum likelihood SNP phylogeny of a cluster of 91 clinical isolates from the youth camp in Belgium, from the UK and from the Netherlands. Each isolate is represented by the year of isolation, the country of isolation (uppercases) and the known recent travel history (capitalized)

Both Public Health England and the National Institute for Public Health and the Environment in the Netherlands were contacted to gain more insights on these isolates and to perform additional analyses on their national *S. sonnei* collections. Figure 3 shows a 25 SNP single-linkage cluster of the Belgian isolates involved in the outbreak (*n* = 9) and isolates from the UK (*n* = 76) and the Netherlands (*n* = 6) isolated between 2011 and 2020 (Online Resource 2) (IQ-TREE, RRID:SCR_017254). The closest isolate to the monophyletic clade formed by the Belgian isolates is a clinical case from England from 2019 at a distance of 3 SNPs. For 39 isolates from both the UK and the Netherlands (42.8%), a recent travel history was reported to Mexico (*n* = 34), to Brazil (*n* = 1), to Costa Rica (*n* = 1), to Curacao (*n* = 1), to USA (*n* = 1) and to mainland Europe (*n* = 1). These isolates are spread throughout the 25 SNP single-linkage cluster. The level of diversity (median SNP distance 25, maximum SNP distance 102) and consistent travel signal suggest that the cases in the youth camp have been exposed to a strain of *S. sonnei* that is endemic in Central America and has been circulating in that region for several decades.

## Conclusions

This local outbreak of *S. sonnei* highlights the power of NGS for outbreak investigations. First, genome analyses enabled internal investigation of the outbreak and were able to cluster the strains of the first camp and link them to the strain isolated in the second camp and to a secondary infection. Secondly, NGS facilitated the further comparison of the outbreak strain with internationally isolated *S. sonnei* strains, highlighting a cluster of closely related strains. Following the collaboration of three European Public Health Institutes, a hypothesis on the origin of the outbreak was formulated. Although travel history is known for only a subset of the isolates that cluster with the outbreak strain, they all point towards recent travels to countries in Central America, such as Mexico, Curacao and Costa Rica [8, 9]. In this particular outbreak, this analysis supports the hypothesis that the strain was introduced to the first camp by an index case who returned ill from a trip to Guatemala and joined the camp, after which the strain was transmitted to other participants by the low hygienic conditions in the sanitary rooms.

## Supplementary information

ESM 1 (XLSX 12 kb)

ESM 2 (XLSX 14.4 kb)

## Data Availability

The datasets generated during and/or analysed during the current study are available in the EMBL Nucleotide Sequence Database (ENA), https://www.ebi.ac.uk/ena/browser/home.
